# Valproic acid inhibits glioblastoma multiforme cell growth via paraoxonase 2 expression

**DOI:** 10.18632/oncotarget.14716

**Published:** 2017-01-18

**Authors:** Jen-Ho Tseng, Cheng-Yi Chen, Pei-Chun Chen, Sheng-Huang Hsiao, Chi-Chen Fan, Yu-Chih Liang, Chie-Pein Chen

**Affiliations:** ^1^ Department of Neurosurgery, Taipei City Hospital, Renai Branch, Taipei 106, Taiwan; ^2^ Graduate Institute of Medical Sciences, College of Medicine, Taipei Medical University, Taipei 110, Taiwan; ^3^ Department of Medical Research, MacKay Memorial Hospital, New Taipei City 251, Taiwan; ^4^ College of Science, National Chengchi University, Taipei 116, Taiwan; ^5^ Department of Physiology, MacKay Memorial Hospital, Taipei 104, Taiwan; ^6^ School of Medical Laboratory Science and Biotechnology, College of Medical Science and Technology, Taipei Medical University, Taipei 110, Taiwan; ^7^ Department of Medicine, Taipei Medical University, Taipei 110, Taiwan

**Keywords:** cell growth, glioblastoma multiforme, histone deacetylase, paraoxonase 2, valproic acid

## Abstract

We studied the potential mechanisms of valproic acid (VPA) in the treatment of glioblastoma multiforme (GBM). Using the human U87, GBM8401, and DBTRG-05MG GBM-derived cell lines, VPA at concentrations of 5 to 20 mM induced G2/M cell cycle arrest and increased the production of reactive oxygen species (ROS). Stress-related molecules such as paraoxonase 2 (PON2), cyclin B1, cdc2, and Bcl-xL were downregulated, but p27, p21 and Bim were upregulated by VPA treatment. VPA response element on the *PON2* promoter was localized at position -400/−1. PON2 protein expression was increased in GBM cells compared with normal brain tissue and there was a negative correlation between the expression of PON2 and Bim. These findings were confirmed by the public Bredel GBM microarray (Gene Expression Omnibus accession: GSE2223) and the Cancer Genome Atlas GBM microarray datasets. Overexpression of PON2 in GBM cells significantly decreased intracellular ROS levels, and PON2 expression was decreased after VPA stimulation compared with controls. Bim expression was significantly induced by VPA in GBM cells with PON2 silencing. These observations were further shown in the subcutaneous GBM8401 cell xenograft of BALB/c nude mice. Our results suggest that VPA reduces PON2 expression in GBM cells, which in turn increases ROS production and induces Bim production that inhibits cancer progression via the PON2–Bim cascade.

## INTRODUCTION

Glioblastoma multiforme (GBM) is the most malignant primary brain tumor with infiltrative growth characteristics. The survival of GBM patients is often less than 15 months from diagnosis, with the 5-year survival rate below 10% even after surgery combined with adjuvant radiotherapy and chemotherapy [1, 2]. Therefore, an advanced understanding of the molecular mechanism of GBM progression is essential to improve clinical outcome.

Valproic acid (VPA) has been widely used in seizure prophylaxis after neurosurgery including GBM, and the pharmacokinetics and toxicity of VPA have been well documented for treating epilepsy [3]. VPA was further shown to inhibit histone deacetylase (HDAC) activity causing impaired epigenetic modification and suppressed cell growth [4]. Thereafter, the antitumor effects of VPA were described *in vitro* and in retrospective clinical studies [5–11]. Several studies revealed that VPA sensitized GBM cells to chemotherapy and radiotherapy by increased cell apoptosis, which involved increased p21 expression and cell cycle arrest, suppression of DNA double strand break repair, and activating pro-apoptotic signaling [12–16]. Reactive oxygen species (ROS) involves tumor development. Overproduction of ROS and antioxidant system defect result in DNA repair impairment and gene expression alteration, contributing to the carcinogenesis process [17, 18].

The paraoxonase (PON) family belongs to endogenous free-radical scavenging enzyme system, which consists of *PON1*, *PON2*, and *PON3* [19]. The three highly conserved genes share about 60% to 70% similarity at the amino acid and nucleotide levels, All three PON members possess antioxidant properties, but their tissue distributions and stress responses are different [19–21]. PON1 and PON3 are found mainly in the liver and are associated with high-density lipoprotein and cholesterol levels. PON2 is an intracellular protein that is expressed extensively in thorax and abdomen tissues, skeletal muscle, artery wall cells, and macrophages [22]. Previous studies have shown that people with impaired PON1 function are at increased risk of cancer development [23–25]. Overexpression of PON3 protects cancer cells from mitochondrial superoxide-mediated cell death [26].

In the present study, we observed that VPA decreased PON2 expression in GBM-derived cell lines. Impaired antioxidant genes may be associated with GBM development, and intracellular PON2 may mediate anti-apoptosis and maintain the growth of GBM. We hypothesized that VPA inhibited PON2 in GBM cells and sensitized GBM cells to oxidative damage and cell death. Our results indicate that VPA suppresses cell growth via the PON2–Bim cascade in GBM cells.

## RESULTS

### VPA attenuates GBM cell growth

**Figure 1:**
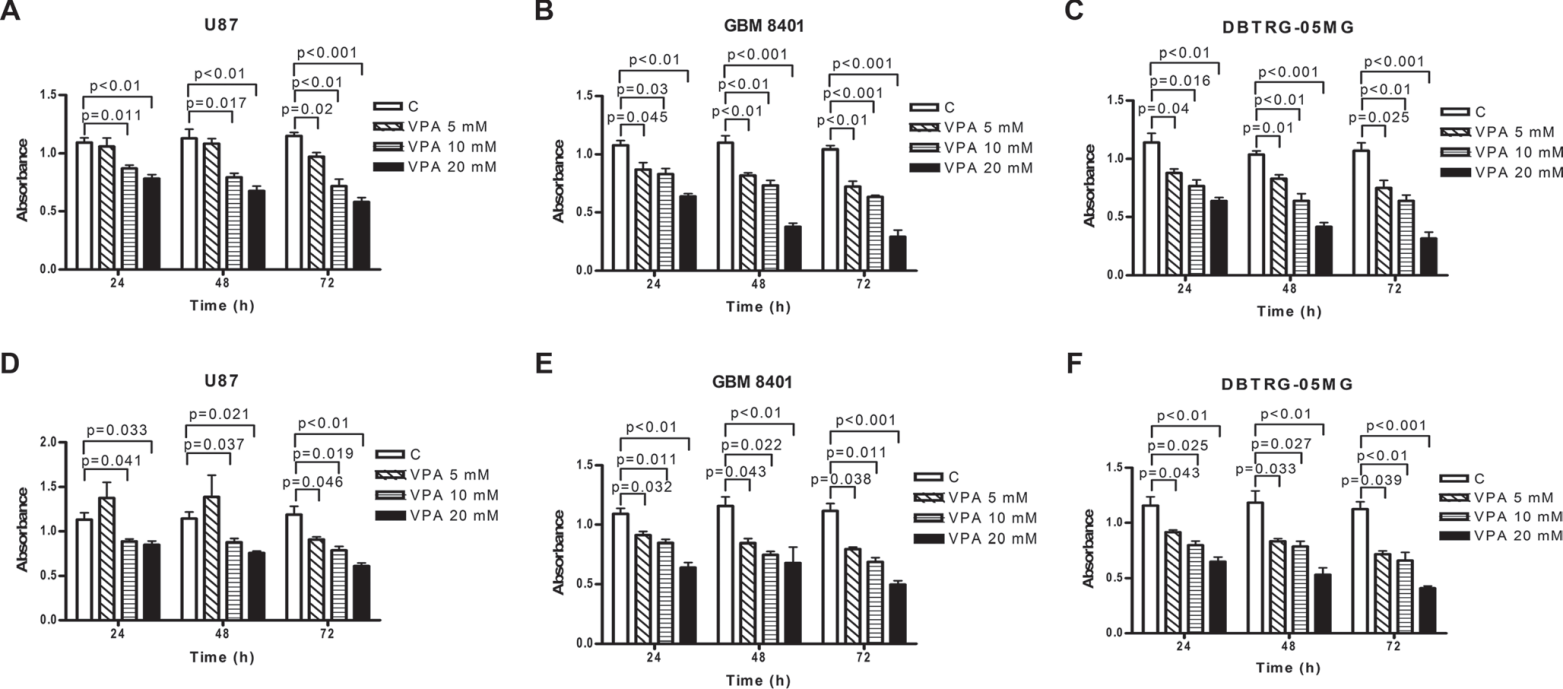
Valproic acid (VPA) inhibits glioblastoma cell growth Cell proliferation was determined in U87 (**A**, **D**), GBM8401 (**B**, **E**), and DBTRG-05MG (**C**, **F**) cells after 5–20 mM VPA stimulation for 24 to 72 h using the MTS (A–C) and Bromodeoxyuridine (BrdU) (D–F) assays. The cell proliferation is significantly decreased in GBM cells using VPA in different doses. The data shown are from three independent experiments performed in triplicate. Error bars: SD. Values are shown as absorbance of VPA-treated cells relative to controls (C; cells without VPA treatment).

**Figure 2:**
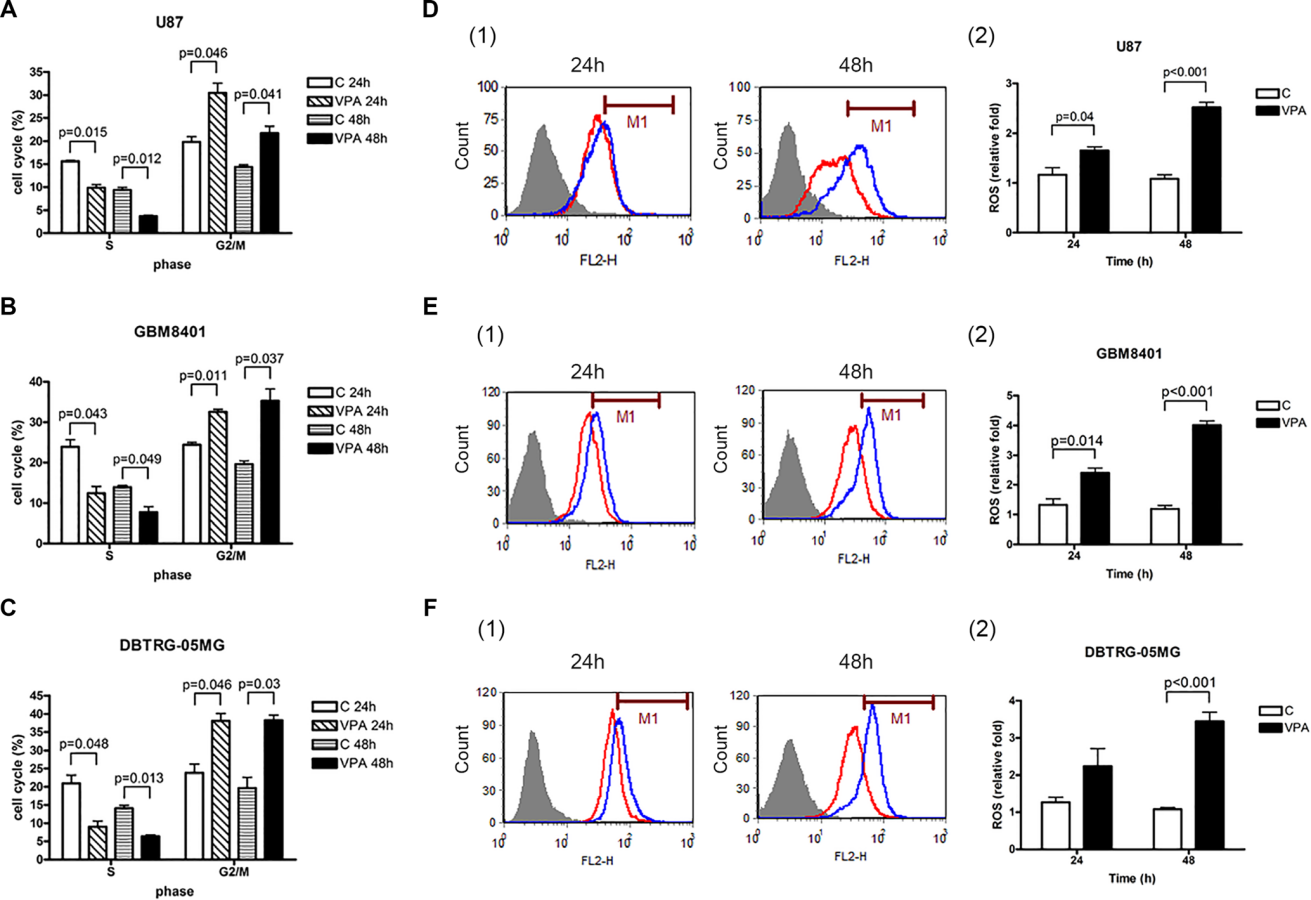
Valproic acid (VPA) induces cell cycle arrest at G2/M phase and increases ROS production The cell cycle was analyzed by flow cytometry in U87 (**A**), GBM8401 (**B**), and DBTRG-05MG (**C**) cells treated with 5 (GBM8401 and DBTRG-05MG) or 10 mM (U87) VPA for 24 to 48 h. The levels of S and G2/M phase are quantified. A significant number of GBM cells arrested at the G2/M phase of cell cycle in GBM cells treated by VPA. The ROS pattern was analyzed by flow cytometry in U87 (**D**), GBM8401 (**E**), and DBTRG-05MG (**F**) cells treated with 5 (GBM8401and DBTRG-05MG) or 10 (U87) mM VPA for 24 to 48 h. The ROS levels are quantified in right panel (2) of D, E, and F, respectively. The ROS level is significantly increased by VPA treatment for 24 to 48 h in GBM cells. The data shown are from three independent experiments. Error bars: SD. Values are shown as cell cycle (%) and relative fold (ROS) of VPA-treated cells relative to controls (cells without VPA treatment). Red line: control; blue line: VPA treatment. C: control.

First, we investigated whether VPA inhibits GBM cell progression. We treated the U87, GBM8401, and DBTRG-05MG GBM cell lines with 5, 10, and 20 mM VPA for 24 to 72 h. Using the MTS and Bromodeoxyuridine (BrdU) assays, the cell growth was reduced significantly by 10 to 20 mM VPA in the U87 cells, and by 5 to 20 mM VPA in the GBM8401 and DBTRG-05MG cells from 24 to 72 h (Figure 1A–1F). Thus, these GBM cells were sensitized with VPA in a time- and dose-dependent manner. Furthermore, to evaluate whether the cell cycle is influenced by VPA, the cell cycle of GBM was assessed by flow cytometry. As expected, the cell cycle was arrested at the G2/M phase at 24 and 48 h in the presence of VPA in U87, GBM8401, and DBTRG-05MG cells, indicating that numbers of GBM cells entering the S phase were significantly reduced (Figure 2A–2C). These observations suggest that VPA decreases cell growth through cell cycle arrest in the G2/M phase in GBM cells.

### VPA increases ROS production

To investigate the mechanism of VPA-mediated cell growth suppression in GBM cells, the effect of VPA on the cell levels of ROS, an important factor in tumor progression [27], was tested on all three GBM-derived cell lines. The ROS level was significantly increased in U87 cells treated with 10 mM VPA for 24 to 48 h as assessed by flow cytometry (Figure 2D). Similarly, significantly increased ROS levels were observed in the GBM8401 and DBTRG-05MG cells after stimulation with 5 mM VPA for 24 to 48 h (Figure 2E, 2F). These results indicate VPA suppresses cell growth via upregulation of ROS production. Additionally, the migration and invasion ability were decreased by VPA in U87, GBM8401 and DBTRG-05MG cells (Supplementary Figure 1A–1F). However, the cell senescence was not altered with VPA by evaluating senescence associated β-galactosidase (SA-βgal) activity [28] in both U87 and GBM8401 cells (Supplementary Figure 1G). We also analyzed the apoptosis characterization. The sub-G1 phase was increased by VPA with PI staining using flow cytometry in U87, GBM8401 and DBTRG-05MG cells (Supplementary Figure 1H).

### VPA influences PON2 and cell cycle–related markers

**Figure 3:**
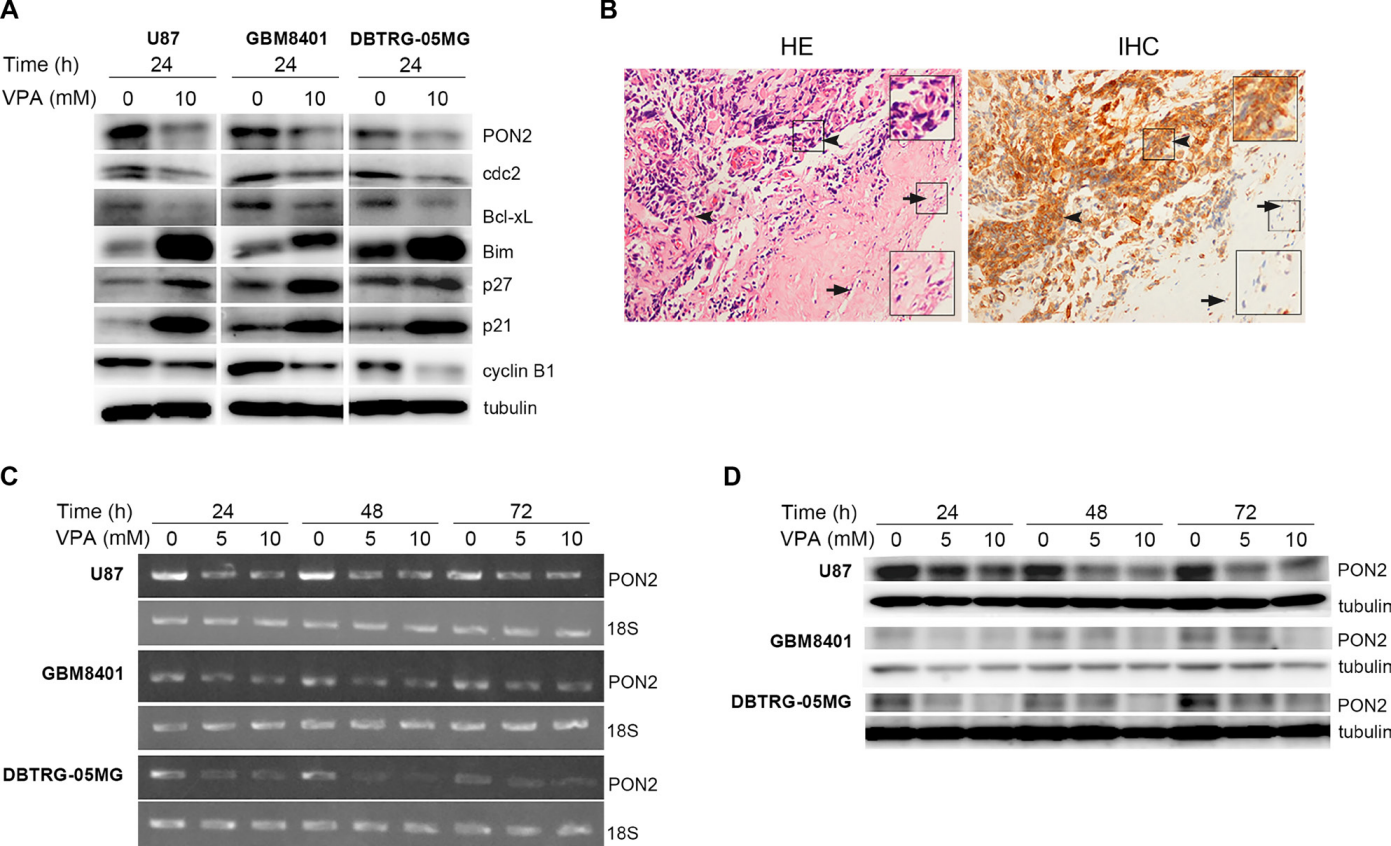
Oxidative stress–related molecules are analyzed in glioblastoma cells (**A**) Western blot to show the expression levels of oxidative stress–related molecules such as PON2, cdc2, Bcl-xL, Bim, p27, p21 and cyclin B1, which were determined after stimulation with valproic acid (VPA) at 24 h in U87, GBM8401, and DBTRG-05MG cell lines. The expression of PON2, cdc2, Bcl-xL and cyclin B1 is decreased, and Bim, p27 and p21 is increased by VPA stimulation in GBM cells. (**B**) Immunohistochemistry (IHC, right panel) to show PON2 protein expression in human GBM biopsies and the left panel is hematoxylin and eosin (HE) staining. The PON2 staining is stronger in tumor cells (arrowhead) compared with normal neuron cells (arrow; magnification: 200×). Large inset: Two-fold magnification of the small inset with arrowheads or arrows. (**C**, **D**) The PON2 mRNA and protein levels were determined after stimulation with 5 and 10 mM VPA for 24 to 72 h in U87, GBM8401, and DBTRG-05MG cell lines by RT-PCR (C) and Western blot (D). The decreased PON2 induced by VPA is in a dose-dependent manner. C: control, cells without VPA treatment.

Based on the above results, we found that cell proliferation and ROS levels were altered by VPA in GBM cells. Therefore, we utilized a commercially available Proteome Profiler Array for cell stress phenotype to evaluate specific VPA-regulated molecules. Several molecules, including PON2 and p27, were identified as potential VPA targets (not shown). The regulation of these molecules was further validated by Western blotting in U87, GBM8401, and DBTRG-05MG cells. PON2 was downregulated and p27 was upregulated at 24 h by VPA (Figure 3A). Since VPA possesses an anti-cancer effect to influence tumor cell proliferation and apoptosis [29, 30], its effect on several additional proliferation and apoptotic-related molecules, such as cyclin B1, cdc2, p21, Bcl-xL, and Bim were investigated. Bim and p21 were upregulated while cyclin B1, cdc2, and Bcl-xL were downregulated at 24 h by VPA treatment in GBM cells (Figure 3A). The results suggest that VPA may regulate these molecules to influence tumor cell proliferation and apoptosis. Since PON2 has been identified as an anti-oxidative protein that decreases intracellular oxidative stress in various cell types [22, 31] and is ubiquitously expressed in a variety of tissues and organs [20], PON2 was selected for further study. Immunohistochemistry of the PON2 expression level in brain tissue of clinical glioblastoma patients revealed that PON2 expression was increased in GBM cells compared with normal brain cells (Figure 3B). We further treated GBM cells with 5 and 10 mM VPA for 24 to 72 h, the PON2 mRNA and protein levels reduced by VPA was in a dose-dependent manner using RT-PCR and Western blot (Figure 3C, 3D).

### VPA inhibits PON2 at the transcriptional level

**Figure 4:**
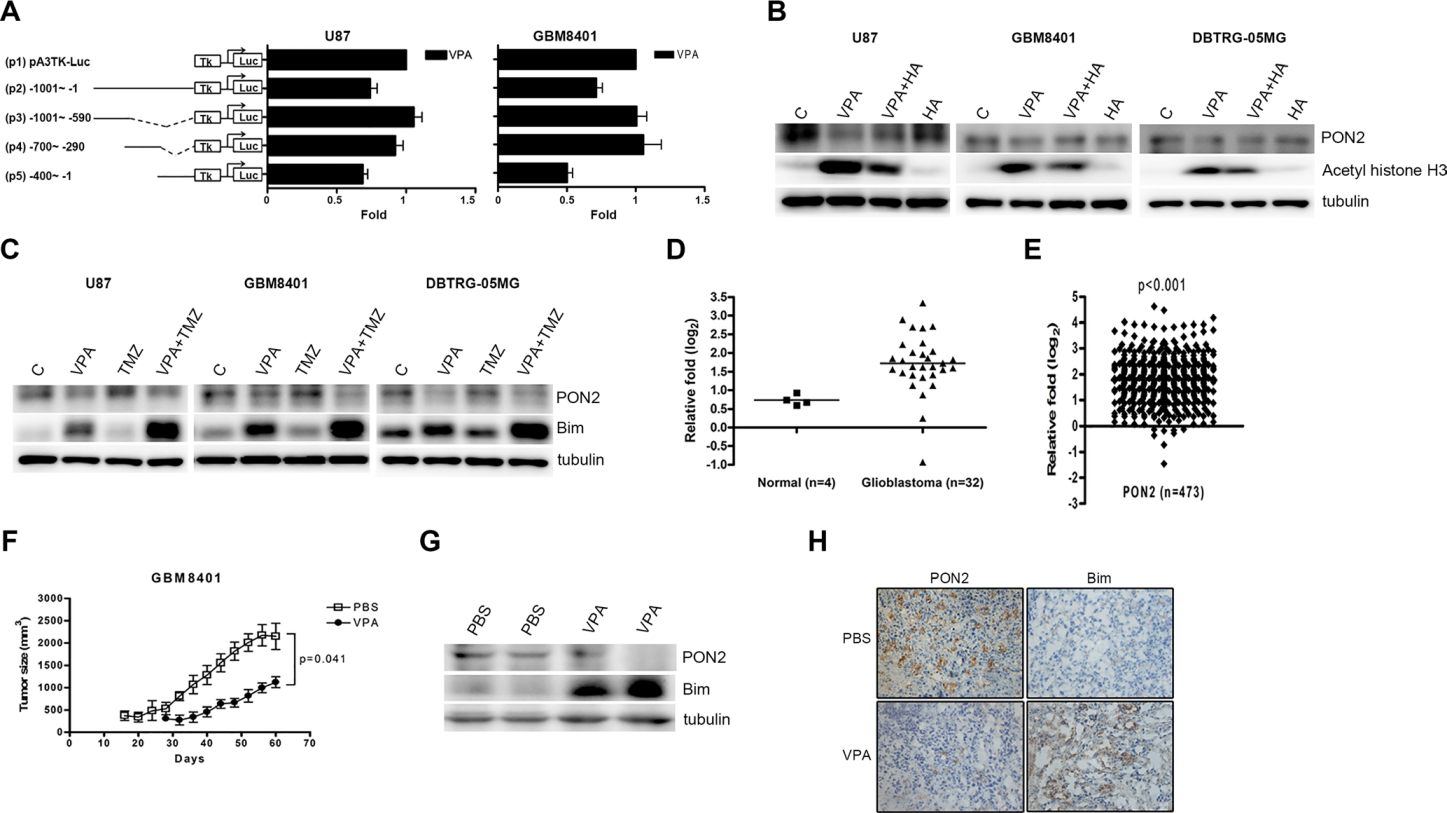
Valproic acid (VPA) inhibits PON2 expression *in vitro* and *in vivo* (**A**) U87 and GBM8401 cells were transfected with the luciferase reporter plasmid driven by the *PON2* 5′-flanking region (position -1001 to -1) with or without pA3TK-luc. Promoter activities were calculated, relative to 0 nM VPA (+VPA/−VPA), and further normalized to the pA3TK-luc control as well as β-galactosidase activity (VPA-induced changes were normalized to that of β-gal). VPA decreases PON2 expression at position -400 to -1. Columns, mean values obtained from at least three independent experiments performed in triplicate; error bars, SE. (**B**) The glioblastoma cells were pre-treated with a HDAC activator, 1-benzoyl-3-phenyl-2-thiourea (HA, 20 μM) for 1 h followed by VPA (U87, 10 mM; GBM8401 and DBTRG-05MG, 5 mM) treatment 24 h. The acetyl histone H3 expression, an acetylation histone protein regulated by HDAC, was not increased as that by VPA. The HA did not significantly alter the PON2 expression compared with control cells. The PON2 expression was downregulated by VPA, which was abrogated by HA in U87, GBM8401 and DBTRG-05MG cells. (**C**) The glioblastoma cells were treated with VPA (U87, 10 mM; GBM8401 and DBTRG-05MG, 5 mM) and temozolomide (TMZ, 40 μM) for 24 hours, which has synergistic effect with VPA on Bim upregulation. PON2 downregulation was not observed in TMZ treatment. TMZ did not have synergistic effect with VPA on PON2 regulation. (**D**, **E**) *PON2* mRNA expression was analyzed in 30 GBM specimens and 4 normal subjects from the Oncomine public Bredel GBM microarray dataset (GEO accession: GSE2223 [38]) (D) and 473 specimens from The Cancer Genome Atlas (TCGA) GBM microarray database (E). The *PON2* level is significantly higher in GBM patients compared with normal controls. (**F**) The nude mice received subcutaneous xenograft with GBM8401 cells (5 × 10^7^) were intraperitoneal injections of PBS (*n* = 3) or VPA (400 mg/kg; *n* = 3) every two days for 60 days. The tumor sizes were measured up to 60 days after inoculation of tumor cells. VPA decreased glioblastoma cells proliferation significantly *in vivo*. (**G**, **H**) The PON2 and Bim levels were measured in tumors of PBS and VPA conditions with GBM8401 cells inoculation by Western blot (G) and immunohistochemistry (H). The PON2 was downregulated and Bim upregulated by VPA in GBM8401 cells-injected mice. C: control, cells without treatment. p: promoter.

Next, we investigated the PON2 regulation mechanism by VPA. The reporter assay was performed to determine the position and clarify the regulatory effects of VPA on *PON2* at the transcriptional level. The *PON2* 5′-flanking region encompassing nucleotides -1000/−1 (relative to the transcription initiation site) (Figure 4A) was cloned and inserted upstream of the luciferase reporter gene in pA3TK-luc (construct p1) to generate construct p2. The pA3TK-luc construct contained a minimum thymidine kinase promoter. Serial deletion fragments were additionally constructed (Figure 4A). The transcriptional activities of the *PON2* promoter fragments are illustrated in Figure 4A. Among these, only the p5 construct was repressed about 2-fold by VPA in U87 and GBM8401 cells (Figure 4A). These findings suggest that VPA inhibits *PON2* at the transcriptional level between position -400/−1 (p5) in glioblastoma.

### VPA attenuates glioblastoma growth via PON2 regulation

VPA has been shown to possess HDAC inhibitor activity [11], hence we investigated whether the VPA-regulated PON2 is affected by HDAC. HDAC decreased the acetylation of histone H3 [35, 36]. We treated GBM cells with a HDAC activator, 1-benzoyl-3-phenyl-2-thiourea [35, 36], which decreased the expression of acetyl histone H3 in GBM cells. VPA inhibited HDAC that increased the expression of acetyl histone H3 in GBM cells. This effect was attenuated by the simultaneous use of VPA and 1-benzoyl-3-phenyl-2-thiourea in these cells. The 1-benzoyl-3-phenyl-2-thiourea did not significantly alter the PON2 expression compared with control cells. The PON2 expression was downregulated by VPA, which was reversed by 1-benzoyl-3-phenyl-2-thiourea in U87, GBM8401 and DBTRG-05MG cells (Figure 4B). Additionally, VPA has been shown to be an effective sensitizing agent in combination with irradiation and chemotherapy to augmentation of therapeutic efficiency on glioblastoma [37]. We stimulated glioblastoma with VPA and temozolomide (TMZ), a DNA alkylating agent, which has synergistic effect with VPA on Bim upregulation (Figure 4C). Because TMZ induced DNA methylation and damage, PON2 downregulation was not observed in TMZ treatment. TMZ did not have synergistic effect with VPA on PON2 regulation (Figure 4C).

The finding was further confirmed by examining the PON2 expression in GBM cells from the public Bredel GBM microarray dataset (Gene Expression Omnibus [GEO] accession: GSE2223 [38]). From the dataset report, the mean *PON2* mRNA level was higher in 30 GBM specimens compared with 4 normal subjects (Figure 4D). Although the public database displayed only 4 normal subjects, the average *PON2* level in glioblastoma patients was about 2.3-fold that of normal subjects (3.75 vs 1.66; Figure 4D). Moreover, this phenomenon was further observed in 473 GBM specimens from The Cancer Genome Atlas (TCGA) GBM microarray database [32] (Figure 4E). To investigate whether the effects of VPA *in vitro* could be applied *in vivo*, we established a subcutaneous xenograft of GBM8401 cells in BALB/c nude mice. Subsequently, nude mice were injected with PBS or VPA (400 mg/kg) intraperitoneally every two days for 60 days [40, 41]. Tumor sizes from the two groups (PBS and VPA) of mice are shown in Figure 4F. The tumor growth of PBS group was initially detected at 14 days, but in VPA group was about 30 days. Tumors sizes of mice injected with PBS were two-fold larger than those of VPA-injected mice (Figure 4F). VPA can inhibit glioblastoma growth *in vivo*. In the study of mouse xenograft, the PON2 expression was downregulated and Bim expression upregulated by VPA in tumors of GBM8401 cells as shown by Western blotting and immunohistochemistry (Figure 4G, 4H).

### PON2 decreases ROS production

**Figure 5:**
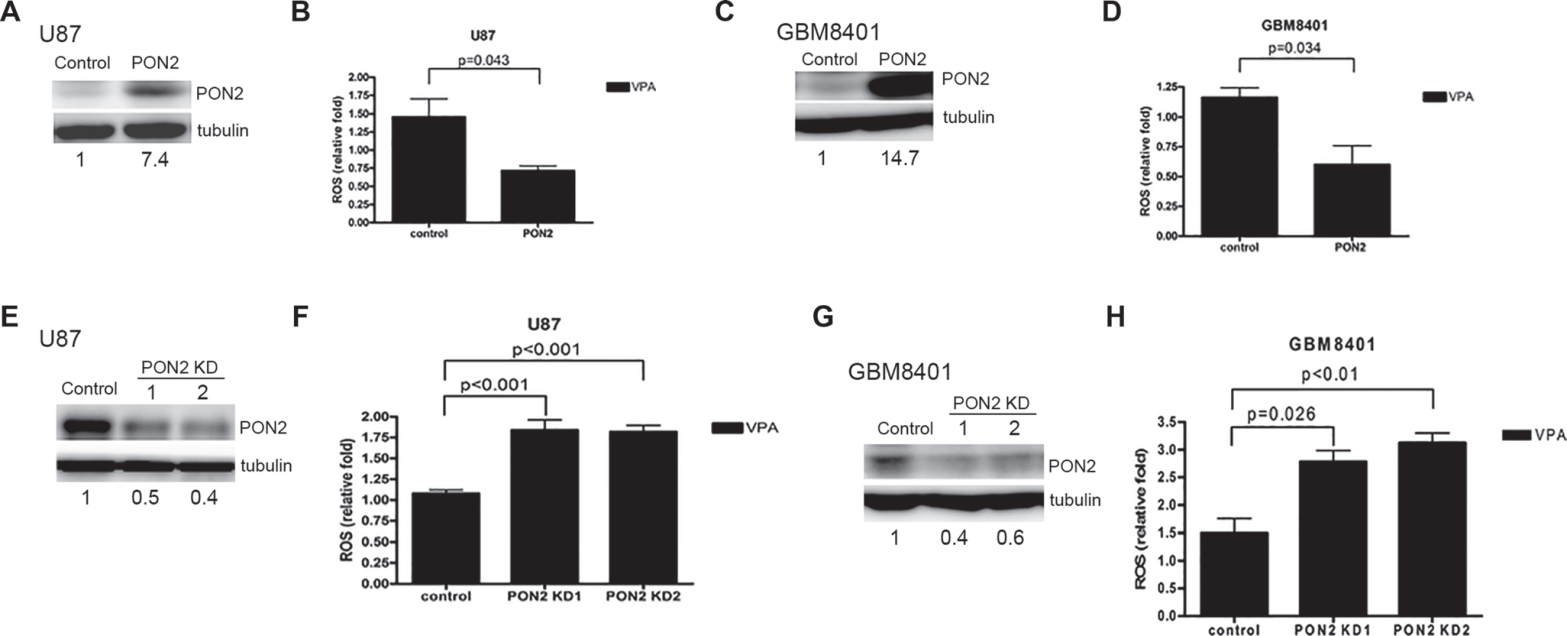
ROS level is inhibited by PON2 Cells overexpressing PON2 (**A**, **C**) and *PON2*-silenced (**E**, **G**) cells were established in U87 (A, E) and GBM8401 (C, G) as shown by Western blot. The ROS levels in U87 (**B**, **F**) and GBM8401 (**D**, **H**) cells in both PON2-overexpressed (B, D) and *PON2*-silenced (F, H) cells were analyzed by flow cytometry. VPA (10 mM) increases the ROS production in vector-control cells, the effect is reduced in PON2-overexpressed cells (B, D). The ROS levels in *PON2*-silenced cells with VPA (10 mM) treatment are higher than that of vector-control cells with VPA treatment (F, H). Control: cells transfected with empty vector only; KD: knockdown.

To determine whether PON2 inhibits ROS production in GBM cells, the ROS level was assessed in PON2-overexpressed GBM cells transiently transfected with *PON2* cDNA. The basal expression level of PON2 in U87, GBM8401, and DBTRG-05MG was shown in Supplemental Figure 2. The expression level of PON2 protein in U87 and GBM8401 cells was increased after PON2 overexpression (Figure 5A, 5C). The ROS level was significantly reduced in PON2-overexpressed cells stimulated with VPA compared with controls in the U87 and GBM8401 cells using flow cytometry (Figure 5B, 5D). Furthermore, we established two transient *PON2*-silenced GBM cells (U87 and GBM8401) in which PON2 protein expression was decreased (Figure 5E, 5G). The ROS level was significantly higher in *PON2*-silenced cells in the presence of VPA compared with controls (Figure 5F, 5H). These findings indicate that PON2 influences ROS production and is implicated in VPA-mediated tumor cell growth arrest in GBM cells.

### VPA increases tumor cell ROS production through PON2–Bim signaling

**Figure 6:**
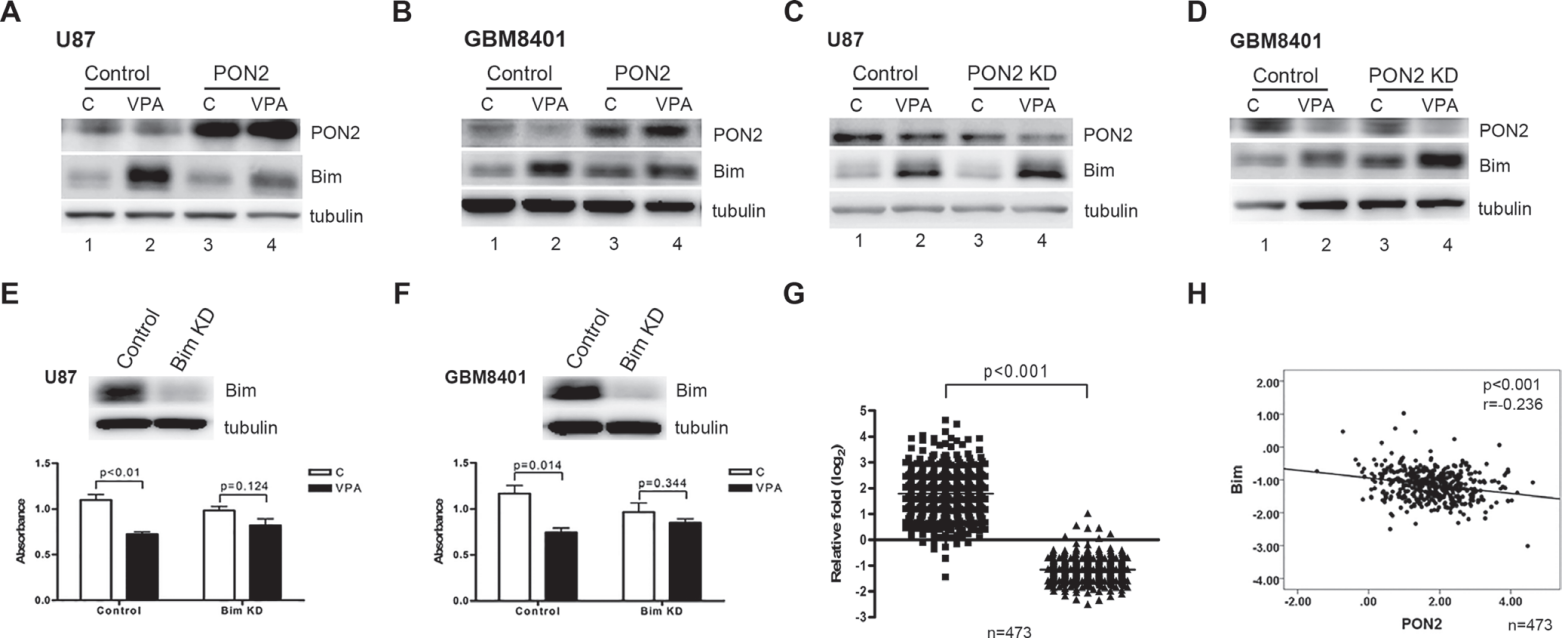
PON2-Bim cascade is involved in VPA-increased ROS The expression levels of PON2 and Bim were determined by Western blot after VPA stimulation in U87 (**A**, **C**) and GBM8401 (**B**, **D**) cells in both PON2-overexpressed (PON2) (A, B) and *PON2*-silenced (PON2 KD) (C, D) conditions. The expression of Bim in VPA treated PON2-overexpressed cells is lower than that of VPA treated vector-control cells (A, B; lanes 2 vs 4). The levels of Bim in VPA-treated *PON2*-silenced cells is higher than that in VPA-treated vector-control cells (C, D; lanes 2 vs 4). (**E**, **F**) The Bim expression and cell proliferation were determined by Western blot and MTS assay after VPA stimulation in U87 and GBM8401 cells with *Bim*-silenced (Bim KD) condition. The decreasing cell proliferation was abrogated in the *Bim*-silenced (Bim KD) condition compared with the vector-control cells in the VPA stimulation cells (**G**, **H**) The mRNA levels of *PON2* and *Bim* were analyzed in 473 specimens from The Cancer Genome Atlas (TCGA) GBM microarray database [32]. The mRNA expression levels of *PON2* and *Bim* in the individuals were significantly different in 473 GBM specimens. The *PON2* level is inversely correlated with *Bim* (Pearson *r* = –0.236, *p <* 0.001). Control: cells transfected with empty vector only; C; cells without VPA treatment; KD: knockdown.

To investigate the downstream signaling pathway involved in PON2-mediated glioblastoma cell growth arrest, we established two transient *PON2*-transfectants overexpressing PON2 in U87 and GBM8401 cells. The expression level of PON2 protein in these transfected U87 and GBM8401 cells was increased compared to levels present in the vector-control cells (Figure 6A, 6B, lanes 1 vs 3). PON2 protein was, however, decreased after stimulation with VPA in the U87 and GBM8401 cells transfected by vector, but not in the PON2 -overexpressed transfectants (Figure 6A, 6B, lanes 1 vs 2, lanes 3 vs 4). We speculate that the expression of PON2 was saturated in PON2-overexpressed cells, hence the PON2 level was not significantly altered after VPA treatment. However, the level of Bim was elevated after VPA treatment (Figure 6A, 6B, lanes 1 vs 2, lanes 3 vs 4), and the expression level was attenuated in the PON2-overexpressed cells with VPA treatment compared with controls (Figure 6A, 6B, lanes 2 vs 4). Similar effects were observed in *PON2*-silenced U87 and GBM8401 cells. The expression level of PON2 protein in U87 and GBM8401 cells was silenced (Figure 6C, 6D, lanes 1 vs 3). Furthermore, the expression of PON2 was decreased after VPA stimulation (Figure 6C, 6D, lanes 1 vs 2, lanes 3 vs 4). Bim expression was activated by VPA in both controls and *PON2*-silenced cells (Figure 6C, 6D, lanes 1 vs 2, lanes 3 vs 4), and the levels were increased in *PON2*-silenced cells compared with controls in U87 and GBM8401 cells (Figure 6C, 6D, lanes 2 vs 4). To investigate whether the Bim regulation is specific in VPA-mediated reducing cell proliferation, we further silenced *Bim* expression in GBM cells. The Bim expression was efficiently suppressed. The decrease in cell proliferation was abrogated in the *Bim*-silenced (Bim KD) condition compared with the vector-control cells in the VPA stimulation in U87 and GBM8401 cells (Figure 6E, 6F). Hence we suggest the Bim is specific in the PON2-Bim pathway of VPA-mediated reducing GBM cell proliferation.

To confirm the clinical significance of the PON2–Bim axis, we determined whether the observed expression values between the PON2 and Bim molecules could be applied in human GBM. The expression levels of the *PON2* and *Bim* genes were retrieved from the published TCGA GBM microarray dataset. The mRNA expression levels of *PON2* and *Bim* in the individuals were significantly different in 473 GBM specimens (Figure 6G), which further revealed *PON2* and *Bim* were significantly inversely correlated (Figure 6H). Taking the data together, we suggest that VPA attenuation of GBM cell proliferation may involve significant upregulation of ROS production via the PON2–Bim cascade.

## DISCUSSION

Here we have shown that cell proliferation is reduced by VPA in U87, GBM8401, and DBTRG-05MG cells. VPA suppresses GBM cell growth via the upregulation of ROS production. *PON2* overexpression and silencing influence ROS production in GBM cells. VPA appears to increase ROS production via the PON2–Bim cascade, leading to cancer cell death.

Our study demonstrated that VPA suppressed the growth of GBM-derived cell lines and induced cell cycle arrest in a time- and dose-dependent manner. ROS level was increased in GBM cells after VPA treatment. Results from protein array analysis revealed the possible mechanisms of inhibitory effects of VPA on GBM cells. These included elevated p27, resulting in cell proliferation suppression and reduced cyclin B1/cdc2, which in turn results in cell cycle arrest at G2/M phase [42], decreased levels of pro-survival Bcl2 and increased pro-apoptotic Bim proteins, which could then result in cell death [43]. Hence, VPA influence may inhibit GBM progression through changes in proliferation and apoptosis.

VPA has been identified as an HDAC inhibitor influencing tumor cell apoptosis, differentiation, and proliferation [29, 30]. Moreover, ROS is an important factor implicated in tumor progression [27]. We also showed that VPA augmented ROS production in GBM cells thus supporting a potential role of VPA in cell growth suppression through the upregulation of ROS production.

A decrease of the anti-oxidant PON2 protein was also detected after using VPA. This deceased PON2 expression could potentiate the cytotoxic effects of ROS and enhance VPA-induced cell cycle arrest. The use of the model of transfectants overexpressing PON2 provided further support for the VPA-induced GBM cell growth suppression being mediated by increased ROS production and that the effect was augmented by decreased PON2.

In a study of non-Hispanic whites, an increased risk of glioma was associated with the single nucleotide polymorphism of *SOD3* [45]. Single nucleotide polymorphisms in *SOD2, SOD3*, *GPX1*, and *NOS1* were found to significantly increase the risk of glioma development in a Chinese population. These data suggest that oxidative stress gene variation might contribute to the etiology of glioma [46]. PON2 was identified in subcellular mitochondrial fractions [31]. PON2 is able to inhibit the generation of mitochondrial superoxide and apoptosis [47]. We observed that GBM cells expressed a higher PON2 protein level compared with normal brain tissue, providing additional support for the importance of PON2 for cancer cell survival.

In agreement with our findings, overexpression of PON2 is observed in solid cancers derived from prostate, liver, pancreas, kidney, lung and thymus [48–50], and T-cell leukemia virus-infected lymphocytes [51]. Kang et al. reported that upregulation of PON2 can be used as a predictor for acute lymphoblastic leukemia with poor prognosis [52]. PON2 accelerates chemoresistance in leukemia cells, and silencing PON2 has resulted in spontaneous apoptosis in various human cancer cells [50]. In keeping with the above observations, our results support PON2 as a tumor promoter via the downregulation of ROS production.

VPA is a relatively weak HDAC inhibitor with activity at millimolar concentrations [53]. The docking and molecular dynamic simulations showed that HDAC inhibited by VPA was through the carboxyl group of VPA coordinating with Zn atom and other local residues (H141-142 and Y360) located at the catalytic site of HDAC. VPA bound with highest affinity at a site located at the acetyl-releasing channel [54]. VPA, either in *in vitro* studies or phase I/II clinical trials, also demonstrated cell growth inhibition effects on both benign cells, such as vascular pericytes, and cancers, such as acute myeloid leukemia and solid malignancies [39, 56]. As the clinical kinetics and bioavailability of VPA are well established, it has promise as an anticancer agent indicated for a reduction of glioma growth either as mono-therapy or as part of a combined treatment. Previous reports have shown that VPA synergistically interacts with chemotherapy or radiotherapy to enhance cytotoxicity in GBM cells by promoting HDAC-dependent transcriptional repression and histone hyperacetylation *in vitro* and *in vivo*, which improves survival in GBM patients [12, 56–62]. Tsai et al. displayed that VPA may be efficacious for GBM with increased histone acetylation, and early administration of VPA for patients within 2 weeks of diagnosis as an adjunct to temozolomide chemotherapy may benefit survival [63]. GBM patients treated with VPA had a significantly longer survival than those who had received other anti-epileptic drugs [11], and VPA may be preferred in patients with glioblastoma who require an anti-epileptic drug during temozolomide-based chemoradiotherapy [37, 64, 65]. For example, patients treated with VPA in combination with temozolomide had a median survival for 69 weeks, longer than those without VPA treatment (61 weeks, *p* = 0.016) [61]. Patients taking VPA had a more favorable median overall survival in (16.9 months), compared to those taking other anti-epileptic drugs (13.6 months, *p* = 0.016) [66].

Our data demonstrate that VPA may suppress GBM cell growth in GBM-derived cell lines through the upregulation of ROS production via the PON2–Bim signaling pathway. In the present study, the selected 5 to 10 mM concentration of VPA (molecular weight 144.21) used in *in vitro* is equivalent to 10-times therapeutic serum level of VPA in human (50–125 μg/mL; the toxic level is greater than 150 μg/mL) [67]. Such a concentration for *in* *vitro* study could be much lower than true therapeutic level in a culture dish, because of the protein binding proportion was neglected. So, effective dose of valproic acid for *in vitro* study may require higher dose to exhibit cytotoxic effects. The higher dose of VPA for *in vivo* study refers to the previous reports [40, 41]. However, humans cannot tolerate high doses of VPA. High-dose VPA experimental data contribute to the clinical efficacy of the drug, which may direct treatments using combination treatment with radiotherapy or chemotherapy, or design other drug formulations in patients. VPA may, thus, represent a potential important therapeutic agent for the treatment for malignant glioma. Further work is needed to study the clinical prognosis of GBM patients treated with VPA to reveal if the PON2 can also be used as a diagnostic marker for predicting therapeutic effect of VPA on GBM patients.

## MATERIALS AND METHODS

### Cell culture

The human glioblastoma cell lines U87, Denver Brain Tumor Research Group 05MG (DBTRG-05MG; ATCC, Manassas, VA) , and GBM8401 (Bioresource Collection Research Center, Hsinchu, Taiwan) were routinely cultured at 37°C in a humidified atmosphere of 95% air and 5% CO_2_ in Dulbecco's modified Eagle's medium (DMEM; Invitrogen, Grand Island, NY) and Roswell Park Memorial Institute 1640 medium (RPMI 1640; Invitrogen) supplemented with 10% fetal bovine serum (Hyclone, Road Logan, UT).

### Cell proliferation assay

Cell proliferation rates were examined using the (3-[4,5-dimethylthiazol-2-yl]-5-[3-carboxymethoxyphenyl]-2-[4-sulfophenyl]-2H-tetrazolium) (MTS) assay (Promega, Madison, WI). Cells (2 × 10^3^) were seeded on 96-well plates overnight. After 6 h starvation, cells were treated with various doses of VPA (2–20 mM) for 48–72 h, and 20 μL MTS solution (10× dilution of 5 mg/mL MTS in DMEM without serum) was added to each well for 3 h at 37°C. Finally, absorbance at 490 nm was measured using a SpectraMax microplate reader.

### BrdU assay

A BrdU incorporation assay was performed using a Cell Proliferation ELISA, BrdU Kit (Roche, Mannheim, Germany). Briefly, the U87, GBM8401 and DBTRG-05MG cells were cultured in 96-well plates at a density of 5000 cells/100 μL/well in a complete growth medium. After stimulating with VPA for 24 to 72 h, the cells were labelled using 100 μL BrdU (1:1000) per well and incubated for 18 h that followed by the procedures as manufacturer's instructions. The reaction was quantified by measuring the absorbance using a scanning multi-well spectrophotometer (Tecan, Mannedorf, Switzerland) at 450 nm, with a reference wavelength of 540 nm.

### Flow cytometry studies on cell cycle and apoptosis

U87, GBM8401, and DBTRG-05MG cells were fixed in ethanol/PBS (7:3, v/v) for 1 h at–20°C. Cells were centrifuged at 3000 rpm for 3 min and pellets were stained with propidium iodide (PI; Sigma-Aldrich, St. Louis, MO) in 250 μL PBS (0.5% Triton X-100) containing 0.125 mg DNase-free RNase A (Roche, South San Francisco, CA) and 6.25 μg PI (Sigma-Aldrich) for 15 min at 4°C in the dark. The cell cycle was examined by flow cytometry. Data were collected and analyzed using FACScan (Becton Dickinson, San Jose, CA) running CellQuest software.

### ROS detection

Cells were trypsinized and resuspended in PBS with RedoxSensor Red CC-1 (Molecular probes, Eugene, OR) for 15 min at room temperature. The ROS level was then determined by flow cytometry using RedoxSensor™ Red CC-1 kit (Molecular Probes) as manufacturer's instructions.

### *In vitro* migration and invasion assays

The influence of VPA on glioblastoma motility was determined as described previously [34]. Briefly, 100 μL cell suspension (1 × 10^5^ cells/ml) was seeded on upper chambers of either non-Matrigel-coated (migration) or Matrigel-coated (invasion; BD Biosciences, San Diego, CA) Transwell with 8-μm-pore size (Falcon BD, Franklin Lakes, New Jersey). The medium in the upper chamber was serum-free medium, whereas the lower chamber medium contained 10% FBS. After incubation for 24 h at 37°C, the cells traversing the filter from the upper to lower chamber were examined via cell counting. Experiments were performed at least three times.

### Cell senescence assay

The senescence assay is to analyze the SA-βgal activity [28]. The U87 and GBM8401 cells (2 × 10^5^ cells) were seeded on 6-well plate. After stimulation with 10 mM VPA for 24 h, cells were washed with PBS twice and fixed with 3.7 % formaldehyde for 10 min at room temperature, followed by staining with SA-βgal staining solution (5 mM potassium ferricyanide, 5 mM potassium ferrocyanide, 150 mM NaCl, 2 mM MgCl_2_ and 1 mg/mL X-gal) overnight at 37°C. The blue SA-βgal positive cells were defined as senescent cells.

### Proteome Profiler™ Array (human cell stress array kit)

U87 cells were either treated with 10 μM VPA for 24 h or left untreated. The cell lysates of U87 were incubated with a Proteome Profiler Array to study cell stress phenotype overnight at 4°C. The array included carbonic anhydrase IX, cited-2, cytochrome C, dickkopf Wnt signaling pathway inhibitor 4, fatty acid binding protein 1, hypoxia-inducible factor 1alpha, heat shock protein (HSP) 60, HSP70, phosphorylated c-Jun N-terminal kinase, nuclear factor–kappaB, p21, p27, PON1, PON2, thioredoxin-1, silent mating type information regulation 2 homolog, and superoxide dismutase 2. The array membrane was washed, followed by incubation with streptavidin-horseradish peroxidase buffer for 30 min at room temperature. Subsequently, the intensity of molecules on the membrane was examined by the chemiluminescence method using a chemiluminescence detection kit (Amersham Biosciences, Piscataway, NJ).

### Western blot analysis

Cells were treated with VPA (5–10 mM), 1-benzoyl-3-phenyl-2-thiourea (HDAC activator, 20 μM, Sigma-Aldrich) and temozolomide (TMZ, 40 μM, Sigma- Aldrich) as the indicated times. Total cell lysate 20-μg proteins were fractionated on 12% sodium dodecyl sulfate-polyacrylamide gel. Separated proteins were transferred onto a nitrocellulose membrane (pH 7.9, Amersham Biosciences), blocked with 5% nonfat powdered milk in PBS, incubated with the specific primary antibodies PON2 (LifeSpan BioSciences, Seattle, WA), Bcl-xL (Santa Cruz Biotechnology, Dallas, TX), Bim, p21, p27, acetyl histone H3 (Cell Signaling, Danvers, MA), cyclin B1, cdc2, and tubulin (Millipore, Billerica, MA) at 4°C overnight, and subsequently hybridized with the appropriate horseradish peroxidase-conjugated secondary antibodies for 1 h at room temperature. Finally, immune complexes were visualized using the chemiluminescence method with a chemiluminescence detection kit (Amersham Biosciences).

### Immunohistochemistry

Usage of archived formalin-fixed, paraffin-embedded tissue block was approved by the Institutional Review Board of MacKay Memorial Hospital. Tissue slides from glioblastoma patients were evaluated by immunohistochemistry and hematoxylin/eosin staining using polyclonal antibody against PON2 (LifeSpan BioSciences) through the avidin-biotin complex method, as described previously [33]. Immunoreactivity for PON2 was visualized using DAB/nickel substrate (Vector Laboratories, Burlingame, CA).

### Reverse transcription polymerase chain reaction (RT-PCR)

Total RNA of glioblastoma cells was extracted using the TRIzol reagent (Life Technologies, Carlsbad, CA) according to the manufacturer's protocol, and cDNA was synthesized using oligodeoxythymidine (Promega, Madison, WI) and a Superscript II reverse transcriptase (Invitrogen). The cDNA was amplified via PCR for 30 cycles at 95°C for 1 min, 58°C for 1 min, and 72°C for 1 min. 18s rRNA was used as an internal control. PCR products were checked by 1% agarose gel (Amresco, Solon, OH) electrophoresis. The primers were displayed in Table 1.

**Table 1:** The primer sequences used for RT-PCR. cloning. siRNA and shRNA

RT-PCR		
Gene	Forward (5′–3′)	Reverse (5′–3′)
PON2	ATGGGGCGGCTGGTGGCT	TTAGAGTTCACAATACAAGGCTCTG
18s rRNA	TAGAGCTAATACATGCCGACGG	GGGCCTCGAAAGAGTCCTGTATT

Gene	Cloning	
PON2 ORF	ATGGGGCGGCTGGTGGCT	TTAGAGTTCACAATACAAGGCTCTG
PON2 P1	TGTTGAAAATTGTCAAGTGTGACCA	CTGCGTCGGGGCCGCGG
PON2 P2	TGTTGAAAATTGTCAAGTGTGACCA	GTGTGTGGAGACCGAGTCTT
PON2 P3	GGCTCATTCCTGTAATTCTAGC	GGAAAGTAACGGGATGCGTC
PON2 P4	AAAAAAAGTATTACGAGGCACAGG	CTGCGTCGGGGCCGCGG

Gene	siRNA SMART pool (5′–3′)	
Bim	UGAGUGUGACCGAGAAGGUAGACAA
	UUGUCUACCUUCUCGGUCACACUCA
	CAUGAGUUGUGACAAAUCAACACAA

Gene	siRNA (5′–3′)	
TRCN0000051643	CCGGGCACATTTCTATGCCACAAATCTCGAGATTTGTGGCATAGAAATGTGCTTTTTG
TRCN0000051644	CCGGGCACTCAGAAATCGACTTAAACTCGAGTTTAAGTCGATTTCTGAGTGCTTTTTG

Abbreviations: ORF: open reading frame; P: promoter.

### Cloning and activities of PON2 promoter fragments

The PON2 promoter (positions -1001 to -1, relative to the transcription initiation site) were ligated into the pA3TK vector, based on the published sequence. Several serial deletion fragments of the PON2 promoter were amplified via PCR and cloned into the pA3TK vector. The constructed promoter sequences were confirmed using automatic DNA sequencing. U87 and GBM8401 cells were cotransfected with 0.3 μg DNA/well of pA3TK vector containing the PON2 promoter sequence and 0.3 μg of SVβ plasmid, a β-galactosidase expression vector (Clontech, Palo Alto, CA), followed by treated with 10 and 5 mM VPA for 24 h in 24-well plates using Lipofectamine 2000 reagent following manufacturer's instructions (Invitrogen) to determine the transcriptional activities of the PON2 promoter. At the end of the treatment period, cells were lysed, luciferase and β-galactosidase activities were measured. Luciferase activity was normalized against that of β-galactosidase. The primers were displayed in Table 1.

### Oncomine^®^ database

Oncomine is a cancer microarray database and integrated data platform available at www.oncomine.org. The Bredel GBM microarray dataset in Oncomine was searched using the criteria of the *PON2* gene and glioblastoma cell.

### TCGA database

TCGA is public and comprehensive databases that consists of various cancer types [32]. We searched the glioblastoma microarray database containing 473 biospecimens from the Data Coordinating Center of TCGA to analyze the *PON2* and *Bim* expression levels.

### Animal study

The BALB/c nude mice (Jackson Immuno Research Laboratories, West Grove, PA) were inoculated subcutaneously with GBM8401 cells (5 × 10^7^ cells) mixed with 100 μl Matrigel ™ (5 mg/mL, BD Biosciences), to enhance tumor growth ability in nude mice [55]. After 1 day of the implantation, VPA (400 mg/kg) [40, 41] or PBS was injected intraperitoneally every two days for 60 days, and PBS group were used as control. Once palpable tumors were established after the inoculation, the tumor xenografts were measured with two dimensions by caliper every two days. These nude mice were sacrificed at 60 days after tumor inoculation. Tumor volume was calculated by the following equation: length × height × width (mm^3^). The Institutional Animal Care and Use Committee of the Mackay Memorial Hospital approved the use of animals in this study.

### Cloning of *PON2*

Total RNA (1 μg) was reverse-transcribed using Superscript II reverse transcriptase (Invitrogen) and oligodeoxythymidine to synthesize template cDNA. The *PON2* cDNA was amplified via a polymerase chain reaction for 30 cycles at 95°C for 1 min, 58°C for 1 min, and 72°C for 1 min. The *PON2* open reading frame was ligated into a pcDNA 3.0 expression vector (Promega) and the resulting construct was sequenced to confirm the presence of the gene. The *PON2* open reading frame primers were displayed in Table 1.

### Establishment of U87 and GBM8401 cell lines transiently overexpressing PON2

The U87 and GBM8401 cells were transiently transfected with the *PON2* cDNA construct in 6-cm cell culture dishes using serum free Opti-MEMi medium and Lipofectamine 2000 reagent following manufacturer's instructions (Invitrogen). After 24 h, the medium of the transfected cells was replaced with regular medium. Subsequently, the expression of PON2 protein in U87 and GBM8401 cells was examined using Western blot analysis. *PON2* cDNA was constructed into pcDNA 3.0 expression vector (Promega), hence, pcDNA 3.0 (Promega) was used as vector control.

### Effect of knockdown Bim and PON2 expression

The siRNA silencing *Bim* was purchased from Invitrogen Corporation. The clone TRCN0000051643 and TRCN0000051644 of short hairpin (sh)RNA targeting *PON2* were purchased from the National RNAi Core Facility (Institute of Molecular Biology, Academia Sinica, Taiwan). The sequence of targeting *PON2* was constructed into pLKO.1 vector (Academia Sinica), which was used as vector control. Transfection of siRNA or shRNA against the endogenous *Bim* and *PON2* genes in U87 and GBM8401 cells were transiently performed using Lipofectamine 2000 reagent (Invitrogen). The expression of Bim and PON2 was confirmed by Western blot analysis. The *Bim* siRNA and *PON2* shRNA sequences were displayed in Table 1.

### Statistical analysis

All values are reported as mean ± SD. Differences were evaluated by Student's *t-test* or Wilcoxon signed rank test, when appropriate. In search for bivariate correlations, this investigation used Pearson's correlation analysis and scatter plot for two continuous variables. *P* < 0.05 was considered significant.

## SUPPLEMENTARY MATERIALS FIGURES



## Abbreviations

Bcl2: B-cell lymphoma 2
BrdU: Bromodeoxyuridine
DMEM: Dulbecco's modified Eagle's medium
DBTRG-05MG: Denver Brain Tumor Research Group 05MG
GBM: glioblastoma multiforme
GEO: Gene Expression Omnibus
HDAC: histone deacetylase
HSP: heat shock protein
MTS: (3-[4,5-dimethylthiazol-2-yl]-5-[3-carboxymethoxyphenyl]-2-[4-sulfophenyl]-2H-tetrazolium)
PON2: paraoxonase 2
PI: propidium iodide
ROS: reactive oxygen species
sh: short hairpin
TCGA: The Cancer Genome Atlas
VPA: valproic acid
