# Supplementary material for: Valproic acid inhibits glioblastoma multiforme cell growth via paraoxonase 2 expression
Source: Oncotarget. 2017 Jan 18;8(9):14666–79. doi: 10.18632/oncotarget.14716 (PMC5362434; doi:10.18632/oncotarget.14716)
Supplement: Supplementary file 1 [file oncotarget-08-14666-s001.pdf]

Valproic acid inhibits glioblastoma multiforme cell growth via paraoxonase 2 expression

Supplementary Materials

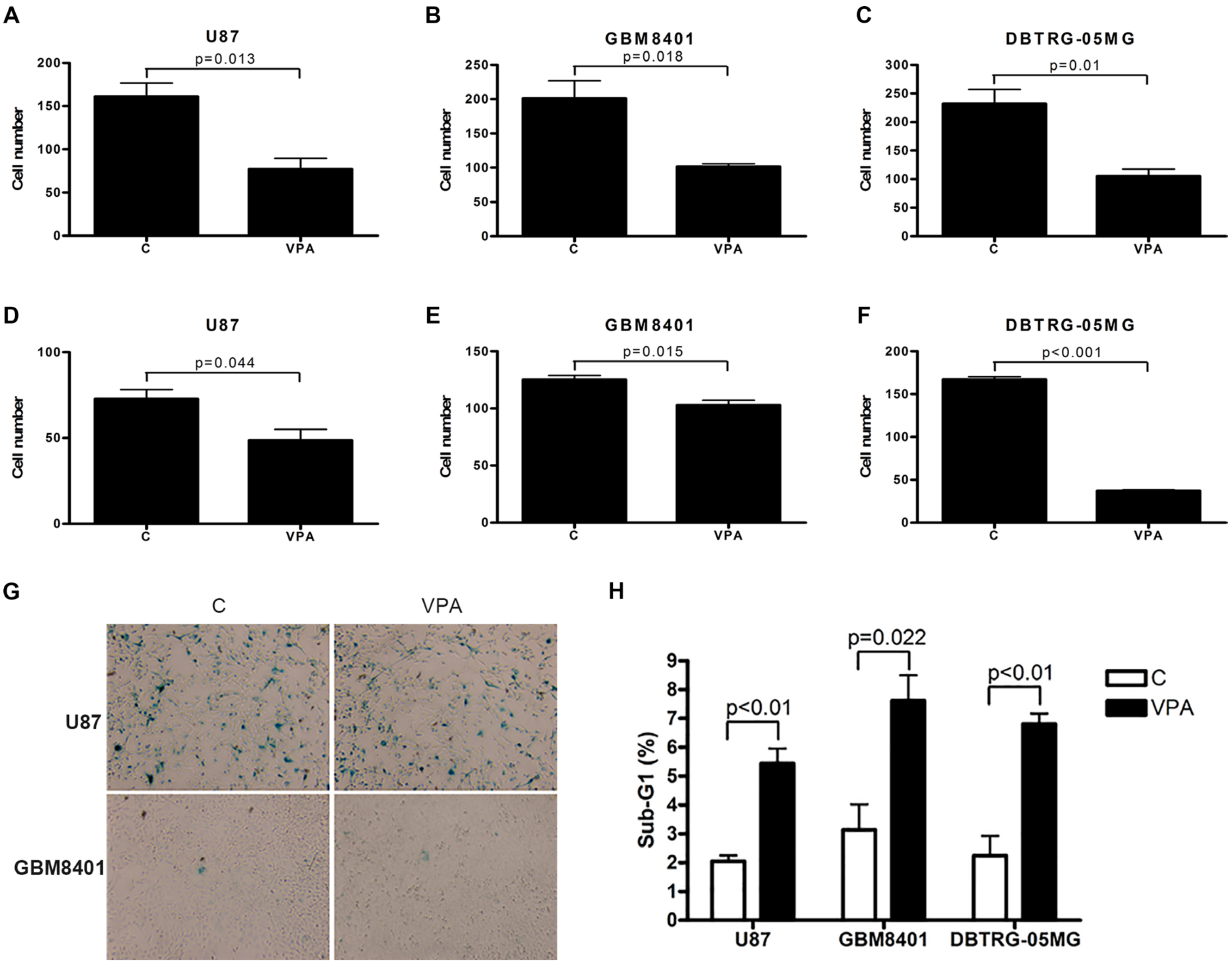

Supplementary Figure 1: The effects of valproic acid (VPA) on glioblastoma. The migration (**A–C**) and invasion (**D–F**) abilities were determined after VPA stimulation in U87 (10 mM, A, D), GBM8401(5 mM, B, E) and DBTRG-05MG (5 mM, C, F) cells for 24 h by a Transwell assay. VPA decreased glioblastoma migration and invasion abilities. (**G**) The glioblastoma senescence was examined. The senescence was not altered by VPA at 24 h in both U87 and GBM8401 cells. (**H**) The apoptosis (sub-G1) was determined after VPA stimulation for 24 h in U87, GBM8401 and DBTRG-05MG cells by flow cytometry. VPA significantly induced glioblastoma apoptosis. C: cells without VPA treatment.

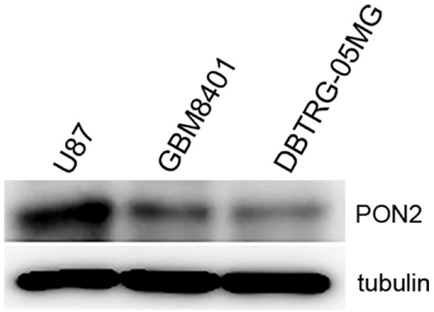

Supplementary Figure 2: The PON2 expression in glioblastoma. The basal level of PON2 was shown in U87, GBM8401 and DBTRG-05MG cells by Western blot.
